# Computer-Aided Structural Diagnosis of Bridges Using Combinations of Static and Dynamic Tests: A Preliminary Investigation

**DOI:** 10.3390/ma16247512

**Published:** 2023-12-05

**Authors:** Tomasz Garbowski, Aram Cornaggia, Maciej Zaborowicz, Sławomir Sowa

**Affiliations:** 1 Department of Biosystems Engineering, Poznań University of Life Sciences, Wojska Polskiego 50, 60-627 Poznań, Poland; maciej.zaborowicz@up.poznan.pl (M.Z.); slawomir.sowa@up.poznan.pl (S.S.); 2 Department of Engineering and Applied Sciences, Università degli Studi di Bergamo, Viale G. Marconi 5, 24044 Dalmine, BG, Italy; aram.cornaggia@unibg.it

**Keywords:** concrete bridge, structural diagnosis, inverse analysis, local minimization algorithm, multistart optimization procedure, trust region algorithm, dynamic modal analysis, finite element method

## Abstract

Reinforced concrete bridges deteriorate over time, therefore displaying a regular need for structural assessment and diagnosis. The reasons for their deterioration are often the following: (a) intensive use, (b) very dynamic loads acting for long periods of time, (c) and sometimes chemical processes that damage the concrete or lead to corrosion of the reinforcement. Assuming the hypothesis that both the stiffness of the material and its density change over time, these parameters shall be identified, preferably in a non-destructive way, in different locations of the investigated structure. Such task is expected to be possibly exerted by means of one or more tests, which must not be laborious or cause the bridge to be out of service for a long time. In this paper, an attempt is made to prepare a procedure based on dynamic tests supplemented with several static measurements, in order to identify the largest number of parameters in the shortest possible time, within an inverse analysis methodology. The proposed procedure employs a popular algorithm for minimizing the objective function, i.e., trust region in the least square framework, as part of the inverse analysis, where the difference between measurements made in situ and those calculated numerically is minimized. As a result of the work performed, optimal sets of measurements and test configurations are proposed, allowing the searched parameters to be found in a reliable manner, with the greatest possible precision.

## 1. Introduction

In current Structural Engineering, growing research and application trends point toward the diagnostic identification and assessment of structures and infrastructures, for characterization and monitoring aims [1,2]. Within such a broad field, particular interest is devoted to bridge structures, for their key infrastructural role and, frequently, historical value [3,4,5]. Recent applications can be found in the literature based on various methodologies and goals, such as employment of Artificial Intelligence [6,7], selection of measurement systems and sensors [8,9,10], damage detection [11,12,13], and, specifically, for reinforced concrete bridges [14,15,16,17].

Proper assessment and monitoring of reinforced concrete bridges, toward diagnosis and prognosis aims, requires a detailed knowledge of the material mechanical properties of the investigated structure, in case of either unknown design data or deteriorated material conditions. Examples of frequent damage scenarios for reinforced concrete bridges can be observed by environmental conditions (see, e.g., [18]), chemical processes (see, e.g., [19,20]) and dynamic loading conditions (see, e.g., [21]). Consequently, modern trends in engineering research and applications exhibit growing interest in efficient and optimized structural testing, supported by the robust calibration of a numerical model for prognosis purposes [22,23].

Toward testing and monitoring aims, several classes of experimental techniques appear to be available, namely in static loading approaches (see, e.g., [24,25]), dynamic Experimental Modal Analysis (EMA; see, e.g., [26,27,28]), and dynamic Operational Modal Analysis (OMA; see, e.g., [29,30,31,32,33,34]). While static testing displays more classic consolidated and robust methods, EMA and OMA testing exhibit significant advantages in analyzing the dynamic properties and behavior of the investigated structure. On the contrary, although advantageously reliable, static testing and EMA require controlled loading conditions, while OMA approaches allow for testing in operational conditions, exploiting infrastructural and ambient loading conditions. In particular, in the state of the art for the structural assessment of reinforced concrete bridges, supported by the above-mentioned approaches, contributions can be found toward specific aims, namely for monitoring and processing techniques (see, e.g., [35,36]), for selection and placement of sensor instrumentation (see, e.g., [37]), and for technical features such as cracking detection (see, e.g., [38]) and prestressing conditions (see, e.g., [39,40,41,42]).

In current and growing engineering research and practice, the above-mentioned experimental methods may be efficiently fostered by structural modeling and identification, particularly within the inverse analysis methodology, for parameter identification and model calibration (see, e.g., [43,44,45,46,47]). Numerous approaches and applications can be found, providing reliable and robust solutions for material characterization, both from a Solid Mechanics standpoint, with regards, e.g., to concrete [48,49], metallic materials [50,51,52,53,54] and composites [55,56], and from a Structural Mechanics point of view, namely assessing buildings and infrastructures [57,58,59,60,61], with growing interest, with reference to the latter, particularly, in Structural Dynamics [62,63,64].

Inverse analyses, specifically applied to parameter identification, in the framework of material characterization, can be described as general methodologies, though based on a detailed sequence of stages, aiming at conceiving a robust and reliable computational procedure. To this aim, the adoption of an inverse analysis approach, in the context of structural diagnosis, as here proposed for bridge-like structures, appears of significant interest, with promising features, also in view of automatized identification and monitoring system.

The expected stages of a complete inverse analysis methodology are briefly described here, in the following, while detailed information can be found in the literature available references (see, e.g., [43,46,65]). An initial selection of the parameters to be estimated in the investigated problem, as sought variables to be calibrated in the devised model, is considered as the first design stage of an inverse approach. Consistently, an experimental procedure may be selected as a physical source of measurement data, namely as an input measurement system setup. A compound test simulation, by means of computational tools (e.g., finite element method, as adopted in the current work), is then developed in order to mimic the experimental testing, specifically to build a model suitable at providing numerical counterparts of the experimental measured quantities. With reference to such a stage, it is worth mentioning that when pseudo-experimental (i.e., numerically generated) data are adopted, in place of experimental data, particularly in devising and validating a new procedure, specific attention should be devoted to the development of the aforementioned numerical model, not to be employed at the same time for data generation and identification, in order to intrinsically avoid biased parameter estimations or “inverse crime conditions”, as referred to in the jargon. In prosecution of the inverse analysis methodology, the definition of a feasible “search domain”, as a space of parameters, from a physical viewpoint, or as an optimization space, from a numerical standpoint, should follow, also being supported by an additional stage based on model sensitivity analysis (see, e.g., [66]), with respect to the sought parameters, driving towards a proper understanding of effective parameter influence and, consistently, the prospected possibility of assessment. Toward completion of the procedure, definition, and minimization of a so called “discrepancy function” is foreseen, in conceiving a suitable scalar norm of the difference between experimental (or pseudo-experimental) measurement data and computed counterparts. Such an optimization task, performed with respect to the sought parameter, and possibly accounting for uncertainties, requires a problem-based selection of a suitable optimization algorithm, capable to tackle the investigated problem with robust tracking, efficient computational cost, and reliable results. Moreover, a final stage of the methodology shall consider validation and accuracy checks, as a required step before actual employment of the devised approach in a diagnostic setup, as here proposed in the aims of the paper.

The paper presents a general procedure, suitable for the identification of material parameters, for, potentially, any concrete bridge structure. The procedure is validated on models with a fairly high level of abstraction, without taking into account many construction details, that may seem naive from the point of view of practical applications, though, besides several simplifications, inspired by classic and reference examples of reinforced concrete bridges [67,68]. However, in the scientific literature, one can often find very practical procedures, based on a simplified engineering approach, which, in the author’s opinion, are on a very similar level of simplification. Moreover, it is worth noting that a limited complexity in the design details of the adopted structural models shall turn out to be advantageous toward a more specific understanding of the effectiveness of the inverse identification devised procedure, as in the aims of the current investigation. Conversely, the simplification hypotheses shall require, in future research and applications, an extension of the methodology, i.e., the adoption of a larger set of sought parameters and details in structural modeling, toward the assessment of technical and design conditions, in bridge structural diagnosis. Although the models presented herein maintain the quality level of measurements as can be obtained in real field tests, with a realistic level of measurement noise, they remain purely pseudo-experimental models. Consistently, with respect to the specific details of every civil engineering structure and infrastructure, the developed method can be used rather for an initial assessment of the optimal set of sensors, their number and best locations, and finally for further developments of a full target inverse procedure based on real experimental data. Despite the simplified engineering approach, the obtained results allow for an optimistic thesis that, at least in the case of the procedure based on pseudo-experimental data, it is always possible to find an optimal testing procedure to identify all the sought parameters, providing that both dynamic and static tests are combined in a proper way. The element of novelty presented in this work is a general procedure for identifying the material parameters of structural elements of various bridges, validated with several pseudo-experimental examples, regarding both conceptual and real models of concrete bridges.

The present paper is organized into five main sections. Following the current introduction, Section 2 is devoted to the presentation of the devised inverse analysis methodology, specifically for the identification procedure and the selected study cases. Consequently, Section 3 describes the gathered results in numerical and graphical forms, to provide a complete overview of the effectiveness of the proposed method. Moreover, Section 4 considers the analysis results toward detailed discussion, in deriving observations, rather to be extended for general validity, on the identification and structural assessment performances. Then, the article proposes a conclusion section, summarizing the main novelties and outcomes, suggesting possible future research developments, further aiming at robust and efficient procedures in structural identification and diagnosis.

## 2. Materials and Methods

### 2.1. Concrete Bridges—Conceptual Examples

**Figure 1:**
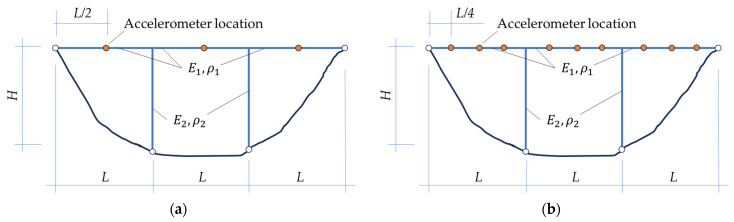
Bridge conceptual examples (where $E_{i}$ and $\rho_{i}$ stand for Young’s moduli and mass densities, respectively, of referred structural elements): (**a**) example 1a, with three deck measurement location points (accelerometers); (**b**) example 1b, with nine deck measurement locations (accelerometers).

**Figure 2:**
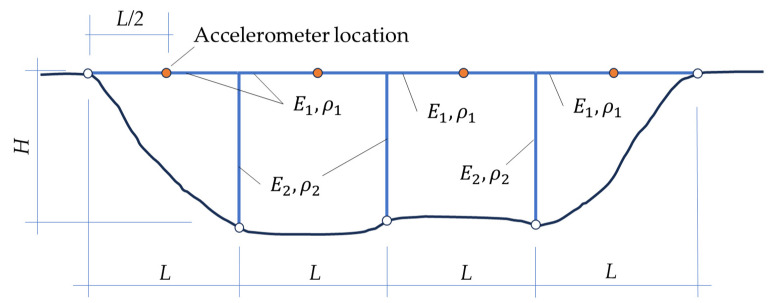
Bridge conceptual example (where $E_{i}$ and $\rho_{i}$ stand for Young’s moduli and mass densities, respectively, of referred structural elements). Example 2 with four measurement location points (accelerometers).

To achieve the aim of material characterization based on experimental measurements of modal properties combined with static load-controlled displacements, in order to illustrate the procedure, two simple examples are here selected, the first one in two configurations of a vertical measurement location point (accelerometer) arrangement (see Figure 1a,b). In both cases, the only parameters that will be identified are Young’s modulus and mass density for the bridge deck and Young’s modulus and mass density of the columns. The second example (see Figure 2) accounts for four deck spans and three columns, while the first one (see Figure 1) considers three deck spans and two columns.

In the first configuration of the arrangement of vertical vibration sensors on the bridge deck (Figure 1a), only three accelerometers are considered at the mid-section of each deck span, while in the second configuration (Figure 1b), nine measurement points are adopted, each one quarter of the deck length apart. In none of the analyzed cases, accelerometers are employed to measure horizontal vibrations on the columns.

It is important to notice that accelerometers are the only source of measurements in this proposed procedure. In fact, the methodology also uses eigenfrequencies, which for a real structure are most often determined using classic, well-consolidated, and/or advanced methods provided by Frequency Domain Decomposition (FDD) [31,32,33,69,70] or Stochastic Subspace Identification (SSI) [71,72,73,74,75,76], while during identification based on a numerical model, these frequencies are determined using dynamic modal analysis.

### 2.2. Inverse Procedure

In the literature on non-destructive methods for the assessment of the technical condition of bridges, one can find articles (see, e.g., [77,78,79]) in which the authors simultaneously determine the stiffness and density of the material in several different parts of the bridge, using dynamic modal properties only, i.e., eigenfrequencies and corresponding eigenmodes. Such a case is also considered in the procedure presented in this paper, but only as one possible methodology variant, due to the fact that, based on identification experience, this option is the least effective. Despite the possibly reduced performance effectiveness of some identification procedures, such as the aforementioned one, the development of such strategies is considered and presented in the current investigation, in order to provide operative guidelines on the weaknesses and strengths and/or on the preferable inverse methodology.

In general, the procedure is tested in four main variants, which use the following:

- variant 1: the discrepancy between the eigenfrequencies of the pseudo-experimental model and the numerical twin only (for few selected first eigenfrequencies);
- variant 2: in addition to the set of natural frequencies, the discrepancy between modes of natural vibration in both models (again, for few selected first modes);
- variant 3: in addition to the set of natural frequencies, the discrepancy in structure deflections in selected locations of the bridge due to static loads in several load cases;
- variant 4: in addition to the set of natural frequencies and eigenmodes, the discrepancy in structure deflections in selected locations of the bridge due to static loads in several load cases.

In all variants, only four parameters are sought, the first pair of parameters for the bridge deck (Young’s modulus and material mass density) and the second pair for the columns; see Equation (1). It is assumed that all elements in a given category have the same parameters (i.e., the material of each column has the same Young’s modulus and density, the same applies to all deck beams, independently from the number of structural components in the presented examples). Consistently, the vector of parameters reads as follows:

(1) $$x=\left[E_{1},\rho_{1},E_{2},\rho_{2}\right],$$

where $E_{1}$ is the Young’s modulus of material used in deck beams, $\rho_{1}$ is the material mass density used in deck beams, $E_{2}$ is the Young’s modulus of material used in columns, and $\rho_{2}$ is the material mass density used in columns.

**Figure 3:**
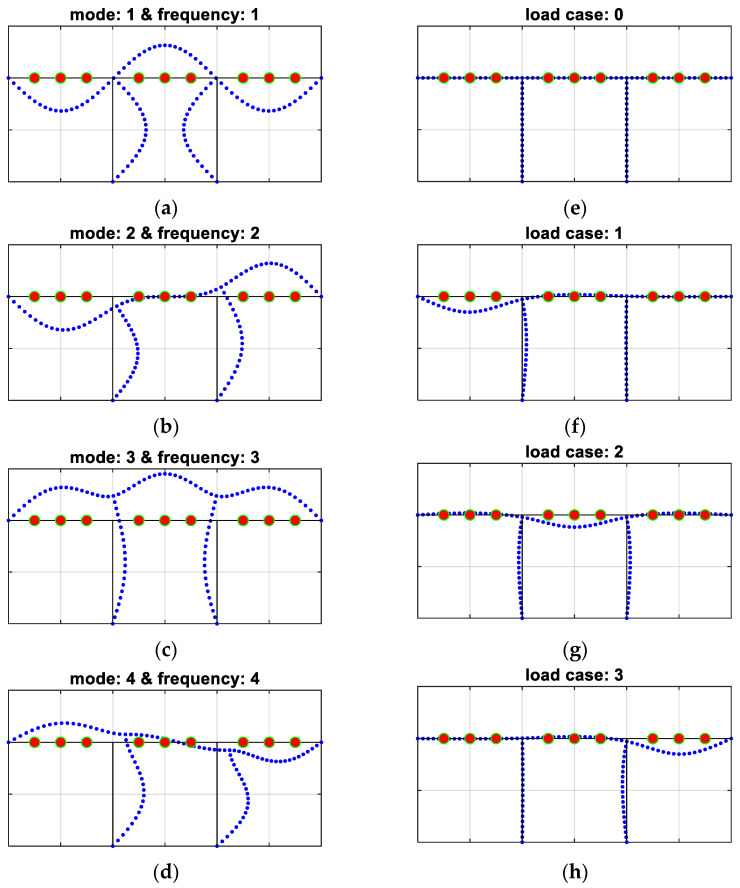
Measured/computed quantities in Example 1b: (**a**–**d**) frequencies and eigenmodes: 1–4; (**e**–**h**) load cases: 0–3.

**Figure 4:**
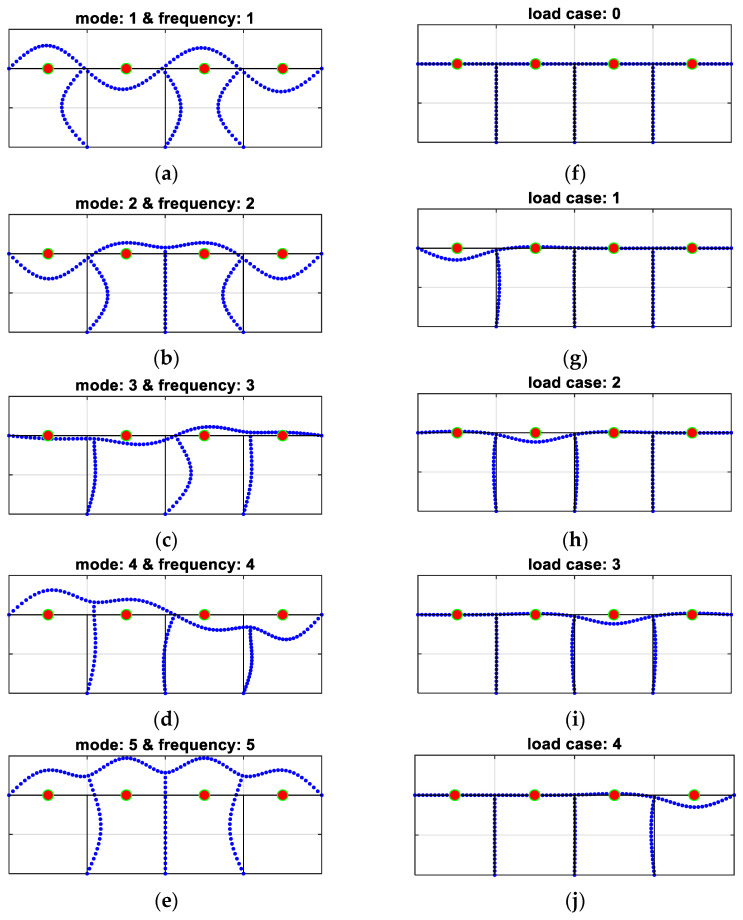
Measured/computed quantities in Example 2: (**a**–**e**) frequencies and eigenmodes: 1–5; (**f**–**j**) load cases: 0–4.

A graphical explanation of all components included in the procedure for different variants is presented in Figure 3 (with reference to Example 1b), where the first four eigenmodes (corresponding to the first four natural frequencies) and four cases of static loads are shown, and in Figure 4 (with reference to Example 2), where the first five eigenmodes (corresponding to the first five natural frequencies) and five cases of static loads are shown. It is important to emphasize that if, for example, the first four eigenfrequencies are used, consistently all four associated eigenvectors are also simultaneously employed in methodology variants 2 and 4. Similarly, for example, for the case described as frequency ID 1…4 and load ID 0, only the first four frequencies (with or without eigenmodes) are used in the inverse procedure together with load case 0 (i.e., displacements measured/computed in accelerometer locations from any static loads are not analyzed). On the contrary, for example, in the cases marked as frequency ID 1…3 and load ID 3, the first three eigenfrequencies (with or without eigenmodes) and three load combinations 1 and 2 and 3 are employed all together in the inverse identification (see Table 1).

### 2.3. Objective Function

In general terms of an inverse analysis problem, since the objective function may consist of more than one measured quantity, which might have completely different orders of magnitude, the scaled differences between the values measured in the pseudo-experimental model and the calculated values in the numerical model are adopted, in order to correctly define the discrepancy terms.

For a first discrepancy term, depending on how many first eigenfrequencies are analyzed, so many weighted differences between the pseudo-experimental model and the computational counterpart create the residual vector in the objective function. Therefore, the *i*-th difference between the measured (pseudo-experimental one, with index *e*) and calculated (numerically, using the finite element method, with index *n*) reads as follows:

(2) $$f_{i}\left(x\right)=1-\frac{f_{i}^{n}\left(x\right)}{f_{i}^{e}},$$

where $f_{i}^{n}(x)$ is an *i*-th frequency numerically computed, while $f_{i}^{e}$ is an *i*-th frequency pseudo-experimentally measured (namely, artificially measured as synthetic data).

In a second discrepancy term, eigenmodes (if they are also analyzed in the selected methodology variant) are represented by measured/calculated values in j=1…R selected measurement locations (namely of accelerometers). Therefore, they create a matrix of residuals for i=1…P frequencies and in j locations. Here, as in the case of the eigenfrequencies, the residual is determined as a scaled difference between the measured and calculated displacement component in the *j*-th location and for the *i*-th frequency, by the following formula:

(3) $$m_{ij}\left(x\right)=1-\frac{m_{ij}^{n}\left(x\right)}{m_{ij}^{e}},$$

where $m_{ij}^{n}(x)$ is an *i*-th mode shape component numerically computed in the *j*-th measurement (accelerometer) location, while $m_{ij}^{e}$ is an *i*-th mode shape component pseudo-experimentally measured in the *j*-th measurement (accelerometer) location.

A third discrepancy term may account for vertical static deflections of the deck (if they are also used in the selected procedure variant), specifically represented by measured/calculated displacements in j=1…R selected locations of measurement points (accelerometers). Similarly, such quantities for eigenmodes create a matrix of residuals for k=1…Q load cases and in j locations. Therefore, the discrepancy term is determined as a scaled difference between the measured and calculated displacement in the *j*-th location and for the *k*-th load case, by the following formula:

(4) $$d_{jk}\left(x\right)=1-\frac{d_{jk}^{n}\left(x\right)}{d_{jk}^{e}},$$

where $d_{jk}^{n}\left(x\right)$ is a vertical displacement computed in the *j*-th measurement location point (accelerometer) for the *k*-th load case, while $d_{jk}^{e}$ is a vertical displacement measured in the *j*-th accelerometer location for the *k*-th load case.

Thus, the objective function consists of the sum of the squared vectors of the differences between the measured and calculated (a) eigenfrequencies (see Equation (2)); (b) eigenmodes (see Equation (3)); and (c) displacement of the structure due to static loads (see Equation (4)).

At the same time, the number of eigenfrequencies, eigenmodes, and load cases is different in each analyzed variant. In addition, not every case uses eigenmodes or static displacements. The objective function, in the full option, is described by the following equation:

(5) $$\omega =f^{T}f+m^{T}m+d^{T}d,$$

where f is an eigenfrequency differences vector:

(6) $$f={\left[f_{1},f_{2},\ldots ,f_{P}\right]}^{T},$$

while m is an eigenmode differences vector (reshaped from a matrix form):

(7) $$m={\left[m_{11},m_{12},\ldots ,m_{1R},m_{21},\ldots m_{PR}\right]}^{T},$$

and d is a static displacement differences vector (reshaped from a matrix form):

(8) $$d={\left[d_{11},d_{12},\ldots ,d_{1Q},d_{21},\ldots d_{RQ}\right]}^{T},$$

where P is the total number of frequencies and mode shapes, R is the total number of measurement location points (accelerometers), and Q is the total number of static load combinations.

### 2.4. Minimization Algorithm

In order to avoid inference based on the local identification response, one can use a global optimization procedure based, among others, on evolutionary algorithms such as the Genetic Algorithm (GA), the Particle Swarm Algorithm (PSA), or the Ant Colony Algorithm (ACA) (see, e.g., [80]). As an alternative, simple random search methods such as Monte Carlo (MC) random search can also be used. However, to achieve the present aims, it appears significantly more efficient to use any local gradient-based algorithm, but in a multi-start version. The algorithm, adopted in the present investigation, creates a certain area around the current solution point, called the trust region. In this area, the method locally approximates the objective function and then updates the solution towards minimizing this local approximation. It is important that the size of the trust region is appropriately adjusted during the iteration of the algorithm, in order to find the optimal solution. Therefore, there is no need to define a step length in the algorithm, as it is computed using the Hessian matrix and gradient vector, both determined based on the residual vector (described in the previous Section 2.3) and on the Jacobian matrix, which is calculated using the least squares method (see, e.g., [81]).

Another positive feature of this adopted approach is the ability to determine the effectiveness of the algorithm by analyzing how many times out of, for example, one hundred different starting points, the algorithm is able to reach the “global” minimum. If the algorithm has an efficiency close to 100%, it means that the problem is well defined and the function is most likely convex within the given limits of arguments. If, on the contrary, the algorithm converges at a different solution point in the parameter space for each run, it means that either the optimization problem is not well defined and/or the function has many local minima.

Although, for ill-posed inverse problems, regularization can be helpful, since multiple procedure variants are employed in this paper, the inefficiency in converging to the global minimum is not corrected here by any additional regularization methods, while it is tackled only by using another methodology variant that is either well-posed (or at least better posed) or by processing until a satisfactory performance is achieved even for not well-posed inverse problem conditions.

### 2.5. Numerical and Pseudo-Experimental Models

The numerical structural models, for direct and inverse analyses in the present investigation, are self-implemented in a MATLAB 2022a environment [82], with mechanical modeling by finite element method. In all the examples, the structural topology is defined with continuous beams, at deck level, and continuous node connections with columns, while the boundary conditions are selected as hinges. The adopted type of finite elements corresponds to Euler-Bernoulli 2D beams and the computational size of each model is kept limited, though sufficiently refined, with no more than 300 elements and 1000 degrees of freedom, in view of efficient repetitive analyses, namely toward a satisfactory compromise between computational burden and accuracy of the results. The output measurable quantities, namely the terms to be employed in the discrepancy function evaluation, are respectively computed, for natural vibration frequencies and natural vibration mode shapes, by classic dynamic modal analysis (finite element modeling, Euler–Bernoulli beam elements, linear elastic material constitutive behavior, zero damping contribution), while, for displacement components upon static load combinations, by classic static structural analysis (finite element modeling, Euler–Bernoulli beam elements, linear elastic material constitutive behavior, small strain, and small displacement assumptions).

The pseudo-experimental model differs from the computational model not only in the number and location of nodes in the finite element mesh, but also in the accuracy of the obtained results. In order to make the data evaluated using the pseudo-experimental model realistic, all measurable quantities, i.e., eigenfrequencies, eigenmodes, and static displacements, are noised by adding a uniformly and randomly distributed noise of up to ±5% of the specific computed values to the calculated values. Although such an approach may turn out not to be entirely rigorous, from an experimental standpoint, it allows for quick and general data noising, without any need for specific different rules to generate various types of realistically noised measurements. Conversely, in the case of real noised experimental data, various filtering and denoising techniques may be adopted, as available in the relevant literature [83,84,85].

It is worth noting that, in the developed analyses, both the starting points and random noise distributions are changed for each analysis (a minimum of twenty different starting points and minimum of five different distributions of random noise), so that in each case there are at least one hundred results for the single variant of inverse procedure. The numerical noise is added to the pseudo-experimental results, as intended to reflect the actual measurement conditions in which all measurements, in the form of accelerometer readings, calculated eigenfrequencies or eigenmodes, and static displacements, are burdened with a certain uncertainty and errors.

## 3. Results

In the present investigation, three examples are analyzed in two variants: with and without the use of eigenmodes; Examples 1a and 1b are analyzed for five variants of the frequency of natural vibration and four combinations of static loads, while Example 2 is analyzed for four variants of the frequency of natural vibration and five combinations of static loads. In each case, as already mentioned in the previous section, a minimum of one hundred inverse analyses is performed, giving a total of approximately 12,000 analyses. This section presents the complete set of obtained results gathered in a condensed form as figures, with statistical analysis, and tables, with mean values and standard deviations, of each case.

**Figure 5:**
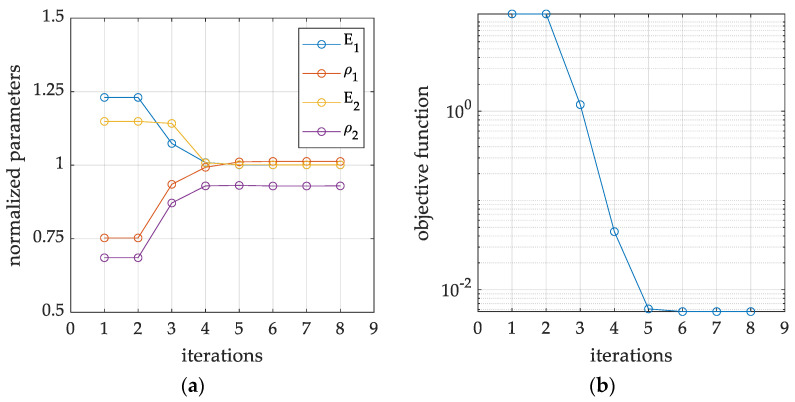
An example of inverse iterative procedure, which convergences to the correct parameters, with reference to Example 1b: (**a**) convergence process in the normalized parameter space; (**b**) iterative minimization of the associated objective function.

**Figure 6:**
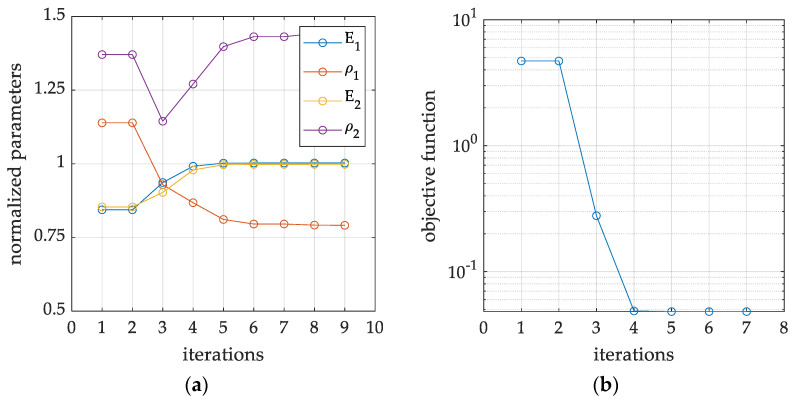
An example of inverse iterative procedure, which convergences to the wrong set of parameters, with reference to Example 2: (**a**) convergence process in the normalized parameter space; (**b**) iterative minimization of the associated objective function.

In particular, Figure 5 and Figure 6 show graphs from a single minimization procedure for a selected example (Example 1b in Figure 5 and Example 2 in Figure 6), specifically: (1) Example 1b with the use of four eigenfrequency values, without the eigenmodes, but with the use of three combinations of static loads and related displacement recorded in nine measurement locations (accelerometers); (2) Example 2, in which four natural frequencies and modes are used, as well as two out of four combinations of static loads in the form of displacements in four measurement point locations (accelerometers). For all cases, it is worth observing that the stopping criterion for the trust region minimization algorithm is set when function convergency is attained to a solution by the solver.

Both figures display the normalized values of the sought parameters (Young’s moduli and mass densities) and the value of the objective function, at each iteration of the inverse procedure. Based on the observation of the objective function values, it can be concluded that in both cases, the minimization algorithm reaches certain minima in the space of the sought parameters, referring to a global minimum in the first case, while to a local minimum only in the second case. Moreover, in the latter case, it can also be clearly observed that the stiffnesses are correctly identified, while the material densities are heavily scaled. Similar conditions can be observed many times, especially when eigenmodes are employed, but without the displacements caused by the static loads.

**Figure 7:**
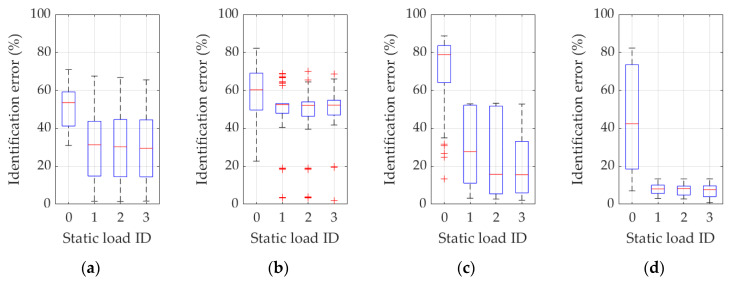
Identification errors for methodology variants 1 and 3 in Example 1a. In addition to the static load combinations, as indicated by ID 0…3 in plots abscissas, the inverse procedure uses (**a**) just the first frequency (frequency ID 1); (**b**) the first and the second frequency (frequency ID 1 and 2); (**c**) the first, the second, and the third frequency (frequency ID 1–3); (**d**) the first four frequencies (frequency ID 1–4).

**Figure 8:**
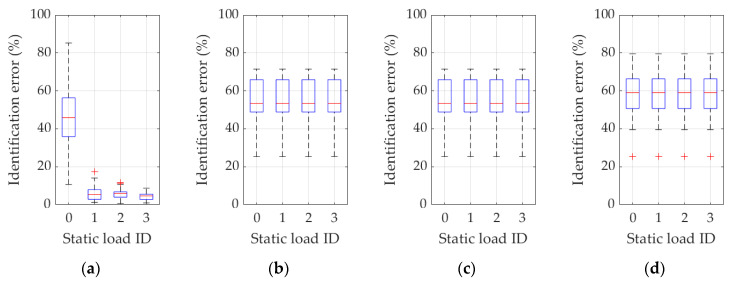
Identification errors for methodology variants 2 and 4 in Example 1a. In addition to the static load combinations, as indicated by ID 0…3 in plots abscissas, the inverse procedure uses (**a**) just the first frequency and eigenmode (frequency ID 1); (**b**) the first and the second frequency and eigenmode (frequency ID 1 and 2); (**c**) the first, the second, and the third frequency and eigenmode (frequency ID 1–3); (**d**) the first four frequencies and eigenmodes (frequency ID 1–4).

**Figure 9:**
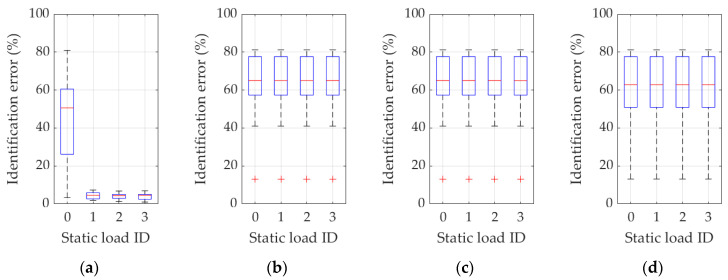
Identification errors for methodology variants 2 and 4 in Example 1b. In addition to the static load combinations, as indicated by ID 0…3 in plots abscissas, the inverse procedure uses (**a**) just the first frequency and eigenmode (frequency ID 1); (**b**) the first and the second frequency and eigenmode (frequency ID 1 and 2); (**c**) the first, the second, and the third frequency and eigenmode (frequency ID 1–3); (**d**) the first four frequencies and eigenmodes (frequency ID 1–4).

**Figure 10:**
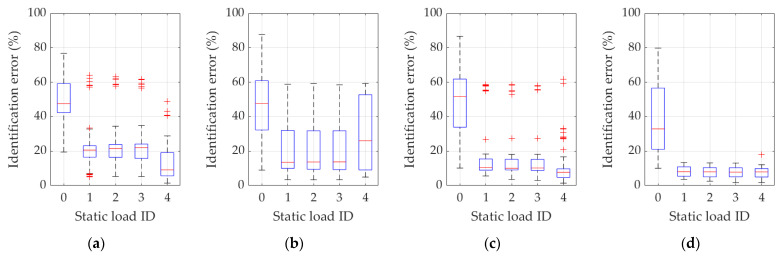
Identification errors for methodology variants 1 and 3 in Example 2. In addition to the static load combinations, as indicated by ID 0…4 in plots abscissas, the inverse procedure uses (**a**) just the first frequency (frequency ID 1); (**b**) the first and the second frequency (frequency ID 1 and 2); (**c**) the first, the second, and the third frequency (frequency ID 1–3); (**d**) the first four frequencies (frequency ID 1–4).

**Figure 11:**
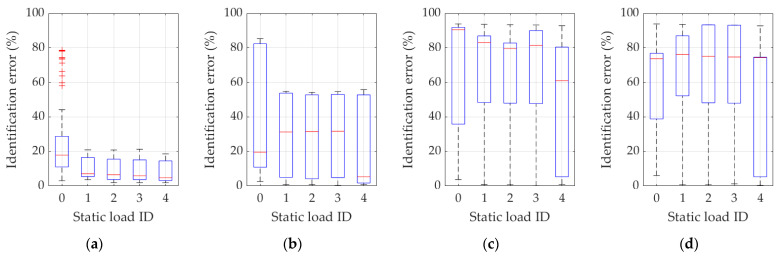
Identification errors for methodology variants 2 and 4 in Example 2. In addition to the static load combinations, as indicated by ID 0…4 in plots abscissas, the inverse procedure uses (**a**) just the first frequency and eigenmode (frequency ID 1); (**b**) the first and second frequency and eigenmode (frequency ID 1 and 2); (**c**) the first, the second, and the third frequency and eigenmode (frequency ID 1–3); (**d**) the first four frequencies and eigenmodes (frequency ID 1–4).

Further results are presented in Figure 7, Figure 8, Figure 9, Figure 10 and Figure 11, showing graphical statistical representations of the obtained results, with a middle mark indicating the median and bottom and top edges of the box indicating the 25th and 75th percentiles, respectively. In addition, whiskers extend to the most extreme data points that are not considered outliers, and outliers are individually plotted with a “+” symbol.

Specifically, Figure 7 displays all results performed for Example 1a with eigenmodes analysis turned off (namely, inverse methodology variants 1 and 3). All selections of eigenfrequency are listed, i.e., when (a) only the first frequency is used (Figure 7a, frequency ID 1); (b) the first and second frequencies (Figure 7b, frequency ID 1 and 2); (c) the first, second, and third frequencies (Figure 7c, frequency ID 1–3); and (d) when the first four frequencies are used (Figure 7d, frequency ID 1–4). The individual boxes in each figure from 7a to 7d are statistical representations of cases where different load combinations are considered (static load combination ID 0…3). Such designation of the load conditions is adopted consistently as summarized in Table 1, so, for example, the load case ID 0 means that no static loads are analyzed, while ID 3 means that all three load cases are considered in combinations, which also means that the displacement values in the measurement location points (accelerometers) are entangled in the inverse procedure function (through Equations (4), (5) and (8)).

In a similar manner, Figure 8 shows all results performed for Example 1a with eigenmodes analysis enabled (namely, for inverse methodology variants 2 and 4). As in the previous figure, all selections of the natural frequency are listed, i.e., when (a) only the first frequency and eigenmode is used (Figure 8a, frequency ID 1); (b) the first and second frequencies and relevant eigenmodes are used (Figure 8b, frequency ID 1 and 2); (c) the first, second, and third frequencies and eigenmodes are used (Figure 8c, frequency ID 1–3); and (d) the first four frequencies and associated eigenmodes are employed (Figure 8d, frequency ID 1–4). The individual boxes in each figure from 8a to 8d propose a statistical representation of the cases where different load combinations are considered (static load combination ID 0…3), newly accordingly to the above-mentioned designation.

Furthermore, Figure 9 collects the results of the statistical analysis for all variants of the procedure for Example 1b with the use of frequencies, eigenmodes, and static load combination displacements, with a graphical representation analogous to the one in Figure 8. Namely, in addition to the employment of displacements from different static load combinations (labeled by ID 0…3), the tested selections read as follows: (a) only the first frequency and eigenmode is used (Figure 9a, frequency ID 1); (b) the first and second frequencies and relevant eigenmodes are considered (Figure 9b, frequency ID 1 and 2); (c) the first, second, and third frequencies and eigenmodes are used (Figure 9c, frequency ID 1–3); (d) the first four frequencies and associated eigenmodes are employed (Figure 9d, frequency ID 1–4).

In turn, Figure 10 and Figure 11 show, similarly to Figure 7 and Figure 8, the results of the statistical analysis of all variants of the procedure for Example 2, based on the use of frequencies (frequency ID 1…4) and static load combination displacements (static load combination ID 0…4) and on the use of frequencies, eigenmodes (frequency ID 1…4), and static load combination displacements (static load combination ID 0…4). Respective results are graphically reported in each figure from 10a to 10d and from 11a to 11d, according to the same ordering definition adopted in the previous figures, as also described in each specific figure caption.

For the sake of completeness in data reporting, Table 2 and Table 3 summarize the results of the statistical analysis of all variants, in terms of identification errors with respect to threshold values. In particular, Table 2 gathers the results relevant to analyses based on the frequency and static displacement discrepancy components (without the use of eigenmodes) – inverse methodology variants 1 and 3, while Table 3 collects the results obtained from analyses produced by tests developed using frequency, eigenmode and static displacement terms in the objective function – inverse procedure variants 2 and 4. Both tables present the mean values of the estimation error along with the standard deviation of each series of analyses, in each variant of the use of frequency (ID 1…6) and static load combinations (ID 0…4). For proper description and understanding of the numerical results, and toward relevant comments proposed in the following Section 4, it is worth noting that all the results in Table 2 and Table 3 are presented in percentages (%), therefore enforcing an improved comparison readability.

## 4. Discussion

Based on the discussed methodology and on the analysis of the complete set of results presented in Figure 7, Figure 8, Figure 9, Figure 10 and Figure 11 and in Table 2 and Table 3, precise and unambiguous comments can be drawn. A first and most important observation indicates that without the use of complemented measurements on displacements resulting from static load cases, practically in no case (regardless of how many natural vibration frequencies and mode shapes are used) a satisfactory result of parameter identification is achieved. In load case ID 0, the error is often over 50%, which practically disqualifies this variant from effective adoption. In detail, one case can be seen (see Figure 11a or Table 3) where only the first frequency (frequency ID 1) and no static load (static load combination ID 0) is used, and yet an average identification error of approximately 24% is obtained. However, Figure 11a also shows that a dozen results oscillate around 60–80% of the error and the standard deviation is over 20%, clearly indicating that there can be no guarantee of obtaining correct results when identifying parameters in real measurement conditions.

A second important observation concerns the use of eigenmodes: the use of only the first eigenmode guarantees satisfactory identification efficiency, provided that at least one variant of the static load combinations is also employed in the procedure. On the contrary, as for a trend highlighted in Figure 8, Figure 9 and Figure 11 and in Table 3 (see, in particular, the raw-wise trend in Table 3), if an increasing number of eigenmodes is employed within the terms of the discrepancy function, a “noising” effect appears, substantially canceling the beneficial effect gathered by the use of static displacement components, namely referring to a possible compensation or not well-posed condition on the discrepancy function.

Furthermore, an additional observation is related to variants in which eigenmodes are not used: in these cases, the use of more eigenfrequencies in the identification process provides better chances for correct results (see, in particular, the improving column-wise trend in Table 2), again provided that the displacements caused by static load are also used in the inverse analysis.

Despite the reduced structural complexity of the analyzed examples, the large number of performed tests allows to attest a general validity and guideline features for the observed trends, and beneficial versus detrimental contributions, in the identification problem, i.e., with reference to the usage of frequencies, eigenmodes, and static load combination displacements, to the amount of measurement data vs. sought parameters and to the selection of optimization tools, in view of an effective and robust inverse procedure.

## 5. Conclusions

From the initially stated goals, the aim of the present work was to propose a procedure that will allow for the most effective assessment of the technical structural conditions of bridges, which have possibly deteriorated over time.

Very simple computational models have been used to achieve this objective, toward a thorough understanding of identification trends and performances, with respect to the available measurement data (natural vibration frequencies, natural vibration mode shapes, displacement components upon static load combinations) and to the validation of the proposed methodology. In the investigation, it has assumed that both the stiffness of the material and its mass density, for various structural components, may change over time; consistently, the proposed procedure indicates the most effective identification strategy to assess these parameters, together with weaknesses and strengths of alternative variant identification paths. The procedure employs non-destructive measurement techniques, as a source of experimental information within the inverse optimization loop, by considering together dynamic operational vibrations and displacement components, from controlled static load testing, in selected locations of the analyzed structure.

The procedure, based on a few simple tests that are not laborious or take the bridge out of service for a long time, relays on dynamic vibration operational measurements supplemented with several static displacement measurements, in order to identify as many constitutive material parameters as possible in the shortest possible time. To minimize the discrepancy function, a popular algorithm for numerical optimization has been used, i.e., the trust region approach in the least squares framework, as a part of the inverse analysis methodology. The main methodological guidelines are extracted, relevant to the beneficial required contribution of static displacement measurements, and to combined effects produced by the use of natural vibration frequencies and/or natural vibration mode shapes. As a result of the work carried out, an optimal set of measurements and test configurations is proposed, allowing to find the sought parameters with the greatest possible precision and, consequently, defining, in a generalized manner, a devised guideline for the suitable selection of the sought parameters, measurement data, and identification strategy, also in more complex inverse applications of structural diagnosis.

The methodology here proposed and validated may be furtherly investigated, in future developments, particularly toward a sequential application of inverse analysis identifications (e.g., for heterogeneous type of measurements), the adoption of Artificial Intelligence in the optimization loops, and the consideration of a larger set of sought parameters, specific for practical, real case, structural diagnosis applications.

## Figures and Tables

**Table 1:** Static loading combinations (load ID) of various load cases adopted in the analyses of Example 1.

Load ID	Load Cases			
	0	1	2	3
0	+			
1		+		
2		+	+	
3		+	+	+

**Table 2:** Percentage mean values and standard deviations of identification errors in the whole set of analysis variants, with the use of frequencies and static load combination displacements, without the use of eigenmodes (inverse procedure variants 1 and 3). Green shading gradient highlights the best performance variants.

Name	Frequency	Load Case				
	ID	0	1	1 and 2	1–3	1–4
Example 1a	1	51.43 ± 11.20	30.28 ± 18.14	30.61 ± 18.46	30.29 ± 18.66	-
	1 and 2	58.96 ± 13.93	49.88 ± 15.52	49.66 ± 15.42	49.77 ± 15.57	-
	1–3	72.28 ± 17.27	28.81 ± 20.76	24.30 ± 20.64	22.93 ± 19.24	-
	1–4	44.36 ± 26.74	7.99 ± 3.02	7.86 ± 3.29	7.24 ± 3.79	-
	1–5	35.15 ± 22.91	7.40 ± 9.42	4.88 ± 2.32	4.64 ± 2.45	-
	1–6	29.23 ± 23.02	7.16 ± 9.25	4.76 ± 2.05	4.54 ± 2.10	-
Example 1b	1	45.44 ± 14.27	16.69 ± 13.62	16.73 ± 13.76	16.65 ± 13.74	-
	1 and 2	41.42 ± 15.24	25.21 ± 14.67	25.13 ± 14.75	24.98 ± 14.81	-
	1–3	40.84 ± 17.58	13.75 ± 5.80	13.72 ± 5.56	13.66 ± 5.71	-
	1–4	30.01 ± 12.94	8.62 ± 4.13	8.48 ± 4.14	8.44 ± 4.06	-
	1–5	24.93 ± 17.50	6.89 ± 3.98	6.75 ± 4.16	6.73 ± 4.19	
Example 2	1	49.55 ± 14.37	21.45 ± 12.06	22.09 ± 12.67	22.30 ± 13.15	13.19 ± 9.69
	1 and 2	47.63 ± 19.87	22.05 ± 16.51	22.05 ± 16.65	21.91 ± 16.76	29.32 ± 20.41
	1–3	48.40 ± 19.61	15.79 ± 14.44	15.55 ± 14.55	15.44 ± 14.70	11.27 ± 11.36
	1–4	39.47 ± 21.38	8.09 ± 3.11	7.71 ± 3.26	7.56 ± 3.28	7.99 ± 4.55

**Table 3:** Percentage mean values and standard deviations of identification errors in the whole set of analysis variants, with the use of frequencies, static load combination displacements, and eigenmodes (inverse procedure variants 2 and 4). Green shading gradient highlights the best performance variants.

Name	Frequency	Load Case				
	ID	0	1	1 and 2	1–3	1–4
Example 1a	1	45.02 ± 15.34	5.68 ± 3.29	5.54 ± 2.56	4.43 ± 2.28	-
	1 and 2	53.76 ± 13.30	53.76 ± 13.30	53.76 ± 13.30	53.76 ± 13.30	-
	1–3	53.76 ± 13.30	53.76 ± 13.30	53.76 ± 13.30	53.76 ± 13.30	-
	1–4	56.83 ± 15.23	56.83 ± 15.23	56.83 ± 15.23	56.83 ± 15.23	-
Example 1b	1	45.06 ± 21.26	4.39 ± 1.63	4.09 ± 1.47	3.96 ± 1.69	-
	1 and 2	61.37 ± 19.94	61.37 ± 19.94	61.37 ± 19.94	61.37 ± 19.94	-
	1–3	61.37 ± 19.94	61.37 ± 19.94	61.37 ± 19.94	61.37 ± 19.94	-
	1–4	59.99 ± 20.14	59.99 ± 30.14	59.99 ± 20.14	59.99 ± 20.14	-
Example 2	1	24.08 ± 20.98	10.09 ± 5.78	8.88 ± 6.04	8.41 ± 5.90	7.74 ± 5.93
	1 and 2	38.62 ± 32.74	29.27 ± 24.88	28.82 ± 24.30	28.95 ± 24.18	23.25 ± 24.65
	1–3	68.09 ± 32.84	67.15 ± 28.42	65.37 ± 27.88	66.08 ± 28.39	52.53 ± 34.02
	1–4	61.54 ± 27.30	69.63 ± 25.35	67.50 ± 24.98	68.38 ± 23.44	53.47 ± 34.49

## Data Availability

Data are contained within the article.

## Nomenclature

**Symbol**	**Description**
x	Vector of sought parameters
$E_{i}$	Young’s modulus of the *i*-th structural component
$\rho_{i}$	Mass density of the *i*-th structural component
$f_{i}$	Discrepancy term for the *i*-th natural vibration frequency
$f_{i}^{n}$	Numerical *i*-th natural vibration frequency
$f_{i}^{e}$	Pseudo-experimental *i*-th natural vibration frequency
$m_{ij}$	Discrepancy term for the *i*-th natural vibration mode shape, at the *j*-th measurement location
$m_{ij}^{n}$	Numerical *j*-th vertical component of the *i*-th natural vibration mode shape
$m_{ij}^{e}$	Pseudo-experimental *j*-th vertical component of the *i*-th natural vibration mode shape
$d_{jk}$	Discrepancy term for the *k*-th static load case, at the *j*-th measurement location
$d_{jk}^{n}$	Numerical *j*-th vertical displacement component for the *k*-th static load case
$d_{jk}^{e}$	Pseudo-experimental *j*-th vertical displacement component for the *k*-th static load case
ω	Discrepancy (or objective) function
f	Discrepancy vector for the natural vibration frequencies
m	Discrepancy vector of the vertical components of the natural vibration mode shapes
d	Discrepancy vector of the vertical displacement components for the static load cases
